# 

**Published:** 1904-08-13

**Authors:** 


**Notices of Births, Deaths, and Marriages** are inserted in this column at a charge of 2s. 6d. for an announcement not exceeding four lines.

**MARRIAGE.**

BEVERIDGE—HAND.—On August 2nd, at Grimsby Parish Church, by the Rev. G. Mahon, Thomas Beveridge, A.I.E.E., etc., Consulting Electrical Engineer, Johannesburg, only surviving son of the late Geo. Beveridge, formerly of Kimberley, to Louisa, second daughter of the late Joseph Hand, of Grimsby. (1624)

**NOTICE TO CORRESPONDENTS.**

All MSS., Letters, Books for Review, and other matters intended for the Editor should be addressed THE EDITOR, "THE HOSPITAL," THE HOSPITAL BUILDINGS, 28 & 29 SOUTHAMPTON STREET, STRAND, W.C.

The Editor cannot undertake to return rejected MS., even when accompanied by stamped directed envelope.

**Notice to Advertisers and Subscribers.**

All Advertisements, Orders for Copies of THE HOSPITAL, and Business Communications should be addressed to **THE MANAGER (Not to the Editor),** THE HOSPITAL, 28 & 29 Southampton Street, Strand, London, W.C.

The Cost of Subscription, post free, is as follows:—Per week, 3½d.; per quarter, 3s. 3d.; per half-year, 6s. 6d.; per annum, 13s. Foreign and Colonial:—Per annum, 19s. 6d., payable, in all cases, in advance.

**NOTICE.—Vol. XXXV. of "The Hospital" (October 1903 to March 1904), in handsome cloth binding, is now on sale at the office of this Journal, price 10s. 6d. Cases for binding the Numbers contained in this Volume 2s. Index to Vol. XXXV., 3½d., post free.**

**The Monthly part for August is now ready. Price 1s., by post, 1s. 3d.**

### BOOKS RECEIVED.

Baillière, Tindall and Cox.

"Practical Chemistry." By P. A. E. Richards.

T. C. and E. C. Jack.
"Entrées of Meat, Game and Poultry, with Sauces."
"Cold Sweets, Jellies and Creams."

J. B. Lippincott and Co.
"International Clinics." Vol. II. XIV. Series.

### MEDICAL APPOINTMENTS.

B. E. G. Bailey, M.R.C.S., L.R.C.P.Lond., Medical Officer of the Midhurst Union. J. J. Blackall, M.D., R.U.I., M.Ch. Certifying Factory Surgeon for the Killadysart District, co. Clare. H. Burton, M.D.Durh., M.R.C.S., Certifying Factory Surgeon for the Marple District, co. Cheshire. Thomas A. Dowse, M.R.C.S., L.R.C.P.Lond., Government Medical Officer at Levuka, Fiji. J. H. Dwan, L.R.C.P. and S.Irel., Certifying Factory Surgeon for the Rathgormick District, co. Waterford. James Graham Forbes, M.A., M.D., D.P.H.Cantab., M.R.C.P.Lond., Assistant Physician to the Metropolitan Hospital. Robert Balfour Graham, F.R.C.S. D.P.H.etc.Edin., Medical Officer of Health for the Burgh of Leven; also Certifying Factory Surgeon for the District of Leven, Largo, and Newburn, under the Factory Acts. W. Sampson Handley, M.S.Lond., F.R.C.S., a Hunterian Professor of Pathology in the Royal College of Surgeons. S. J. J. Kirby, M.D.Brux., L.R.C.P.Edin., M.R.C.S.Eng., Medical Officer of Health for the Mutford and Lothingland Rural District and Oulton Broad Urban District. Ewen J. Maclean, M.D., M.R.C.P.Lond., F.R.S.Edin., Lecturer in Midwifery (under the Midwives Act) to the University College of South Wales and Monmouthshire, Cardiff. D. McEniry, L.R.C.P. and S.Edin., Certifying Factory Surgeon for the Ballamacarbery District, co. Waterford. M. McSwiney, M.B., R.U.I., L.F.P.S.Glasg., Certifying Factory Surgeon for the Johnstown District, co. Cork. H. G. Magrath, L.R.C.P. and S.Edin. L.F.P.S.Glasg., Certifying Factory Surgeon for the Cranborne District, co. Dorset. Charles E. Maguire, M.D., C.M.Aberd., District Medical Officer of Suva. C. D. Outred, M.R.C.S., L.R.C.P.Lond., Medical Officer for the Grays District of the Orsett Union. J. Quirke, L.R.C.P. and S.Irel., Certifying Factory Surgeon for the Piltown District, co. Kilkenny. Harry James Spon, M.R.C.S.Eng., L.R.C.P., D.P.H.Camb., Surgeon to the Surrey Dispensary.

Wigan Infirmary.—The following appointments have been made:—Honorary Medical Officers: John Blair, M.D., R.U.I., A. J. Greene, M.C.R.S., L.R.C.P.Lond., Ferdinand Rees, M.D.Glasg. Honorary Surgeon Dentist: Andrew Thom, L.D.S., R.C.S.Edin.

**Medical Appointments.**

**IMPORTANT.** **NOW READY.**

### NEW EDITION.

### THE NURSE'S PRONOUNCING DICTIONARY

OF

### Medical Terms and Nursing Treatment.

Compiled by HONNOR MORTEN.

**FIFTH EDITION, with Phonetic Pronunciations,**

Revised and Edited by MARY I. BURDETT,

Member of Guy's Hospital Past and Present Nurses' League.

Price **2/-** post free.

THE value of this well-known Dictionary has been greatly enhanced by the many new and important additions to the text, **while the fact that the phonetic pronunciation is now appended to the majority of the words will add immeasurably to the practical utility of this already indispensable Companion to Nurses.**

Price **2/-** post free.

SUITABLE SIZE FOR THE APRON POCKET.

162 pp., cloth, **2/-** post free, or in handsome leather gilt, **2/6** net ; **2/8** post free.

*Of all Booksellers, or of*

THE SCIENTIFIC PRESS, LTD.,

28 & 29 Southampton Street, Strand,

London, W.C.

